# Simon’s Clinical Diagnosis

**Published:** 1898-01

**Authors:** 


SIMON'S CLINICAL DIAGNOSIS. New (2d) edition, revised and enlarged. A manual of clinical diagnosis by microscopical and chemical methods. For students, hospital physicians and practitioners. By CHARLES E. SIMON, M. D., late Assistant Resident Physician Johns Hopkins Hospital, Baltimore. Octavo, 530 pages, with 135 engravings and 14 full-page colored plates. Philadelphia and New York : Lea Brothers & Co. 1897.

Clinical diagnosis, although rarely taught in the medical schools, is so important and necessary to the general practitioner that in the past few years a number of treatises have appeared bearing upon this subject. Two excellent works have appeared of late in America, and the book by Simon holds high rank with the others on the same subject.

No continuing success can be built on empiricism or upon the proportion of guess-work which is inseparable from dependence upon the experienced eye. Diagnosis is now the password of medical science, and the more thorough and exact such diagnoses the more favorable and conscientious the treatment.

The author devotes nearly a hundred pages to the consideration of the blood, describing the various instruments for estimating the number of corpuscles, the amount of hemoglobin and the specific gravity. The microscopy and bacteriology of the blood are also carefully considered. A short chapter on the secretions of the mouth follows, dealing mostly with the condition of the saliva in various forms of stomatitis.

Chapter III. deals with the gastric juice and the gastric contents; Chapter IV. with the feces, first considering the examination of the normal feces, chemically and microscopically, then the pathological conditions, the presence of parasites, as protozoa and vermes and the condition of the feces in the various diseases of the intestinal tract. Chapter V. treats of the nasal secretion ; Chapter VI. of the sputum and Chapter VII. of the urine. The last chapter is gone over exceedingly thorough and complete and is accompanied by several full-page colored plates. Chapter VIII. deals with the transudates and exudates; Chapter IX. with the examination of the cystic contents; Chapter X. with the examination of the cerebro-spinal fluid; Chapter XI. with the semen; Chapter XII. with the vaginal discharge and Chapter XIII. with the secretion of the mammary glands.

The diction is easy and graceful, the descriptions clear and concise and the directions careful and comprehensive. It is a safe guide to follow and should be in the hands of all conscientious physicians. The plates are beautifully executed and the typography of the best. W. C. K.

